# Chemokine CXCL16 mediates acinar cell necrosis in cerulein induced acute pancreatitis in mice

**DOI:** 10.1038/s41598-018-27200-y

**Published:** 2018-06-11

**Authors:** Yojiro Sakuma, Yuzo Kodama, Takaaki Eguchi, Norimitsu Uza, Yoshihisa Tsuji, Masahiro Shiokawa, Takahisa Maruno, Katsutoshi Kuriyama, Yoshihiro Nishikawa, Yuki Yamauchi, Motoyuki Tsuda, Tatsuki Ueda, Tomoaki Matsumori, Toshihiro Morita, Teruko Tomono, Nobuyuki Kakiuchi, Atsushi Mima, Yuko Sogabe, Saiko Marui, Takeshi Kuwada, Akihiko Okada, Tomohiro Watanabe, Hiroshi Nakase, Tsutomu Chiba, Hiroshi Seno

**Affiliations:** 1 Department of Gastroenterology and Hepatology, Graduate School of Medicine, Kyoto University, Kyoto, Japan; 2 Department of Gastroenterology and Hepatology, Saiseikai Nakatsu Hospital, Osaka, Japan; 3 Department of Clinical Education, Shiga University of Medical Science, Otsu, Japan; 4 Department of Gastroenterology and Hepatology, Kindai University Faculty of Medicine, Osaka-Sayama, Japan; 5 Department of Gastroenterology and Hepatology, Sapporo Medical University School of Medicine, Sapporo, Japan; 6 Kansai Electric Power Hospital, Osaka, Japan

## Abstract

Severe acute pancreatitis is a lethal inflammatory disease frequently accompanied by pancreatic necrosis. We aimed to identify a key regulator in the development of pancreatic necrosis. A cytokine/chemokine array using sera from patients with acute pancreatitis (AP) revealed that serum CXCL16 levels were elevated according to the severity of pancreatitis. In a mouse model of AP, Cxcl16 expression was induced in pancreatic acini in the late phase with the development of pancreatic necrosis. *Cxcl16*^−/−^ mice revealed similar sensitivity as *wild-type* (*WT)* mice to the onset of pancreatitis, but better resisted development of acinar cell necrosis with attenuated neutrophil infiltration. A cytokine array and immunohistochemistry revealed lower expression of Ccl9, a neutrophil chemoattractant, in the pancreatic acini of *Cxcl16*^−/−^ mice than *WT* mice. *Ccl9* mRNA expression was induced by stimulation with Cxcl16 protein in pancreatic acinar cells *in vitro*, suggesting a Cxcl16/Ccl9 cascade. Neutralizing antibody against Cxcl16 ameliorated pancreatic injury in the mouse AP model with decreased Ccl9 expression and less neutrophil accumulation. In conclusion, Cxcl16 expressed in pancreatic acini contributes to the development of acinar cell necrosis through the induction of Ccl9 and subsequent neutrophil infiltration. CXCL16 could be a new therapeutic target in AP.

## Introduction

Severe acute pancreatitis (SAP) is a life-threatening systemic inflammatory disease with a 30% mortality rate^1,2^. The development of SAP is frequently accompanied by underlying pancreatic necrosis. Although edematous pancreatitis is a self-limiting disease treated by supportive management and therefore regarded as mild acute pancreatitis (MAP), pancreatic necrosis can provoke local infection and subsequent systemic inflammatory reactions characterized by enhanced cytokine responses: also called systemic inflammatory response syndrome or multiple organ dysfunction syndromes. To improve the outcome of patients with SAP, it is necessary to understand molecular mechanisms distinguishing necrotizing pancreatitis from edematous pancreatitis.

Given the fact that pathological analysis utilizing surgically resected tissue samples of necrotizing pancreatitis revealed marked infiltration of immune cells^3^, immune cells are considered to play a pivotal role in the development of pancreatic necrosis^4,5^. Indeed, leukocyte depletion ameliorates pancreatic injury in mouse acute pancreatitis (AP) models^6,7^. It is now generally accepted that the development of pancreatitis depends upon pro-inflammatory cytokine responses secreted by leukocytes accumulated into the inflamed pancreas^8,9^. Such pro-inflammatory cytokines produced by leukocytes include tumor necrosis factor α (TNFα) and interleukin 1β (IL1β), both of which mediate early-phase inflammation during AP. In addition, IL6 is regarded as a late-phase cytokine during AP^10–12^. As for the chemokines that recruit leukocytes into pancreatic tissue, there are some reports showing the importance of CCL2 secreted by acinar cells in the early phase of AP^13–16^. However, the key chemokine responses in the progression of necrotizing pancreatitis, especially at a late phase, have not yet been identified.

CXCL16, a CXC chemokine, was first reported to be overexpressed in chronic pancreatitis and pancreatic cancer^17^, and recently reported to be an indicator of necrotizing pancreatitis^18^. Among all the chemokines, only CXCL16/CXCR6 and CX3CL1/CX3CR1 are the ligand/receptor sets that bind one-to-one to each other, while other chemokines share some receptors^19,20^. The unique characteristics of CXCL16 are reported to be involved in both innate immunity and Th1-acquired immune responses. In innate immunity, the transmembrane form of CXCL16 on the surface of antigen presenting cells mediates phagocytosis as a scavenger receptor^21^. In Th1-acquired immune responses, CXCL16 functions as a co-stimulatory molecule and its soluble form is a chemoattractant for activated T-cells^22–24^. In addition to these functions in innate and acquired immunity, CXCL16 can induce downstream gene expressions through various signaling cascades^25–27^. For example, IL6 expression is upregulated by CXCL16/CXCR6 signaling in human prostate cancer cells^25^. How CXCL16 affects the development of necrotizing pancreatitis through its multiple functions, however, remains unclear.

Here we provide the evidence that the chemokine CXCL16 is related to the progression of human necrotizing pancreatitis. Using a mouse model, we found that Cxcl16 is produced by pancreatic acinar cells in the late phase of AP and then accelerates inflammatory cascades through the induction of Ccl9 in an autocrine manner. We also propose a potential treatment approach against AP by inhibiting Cxcl16-mediated signaling pathways.

## Materials and Methods

### Cytokine/chemokine array with human serum samples

Human serum samples were obtained from patients with AP admitted to Kyoto University Hospital from 2000 to 2015, and from healthy donors. Serum samples were obtained at the time of grading severity of pancreatitis, and stored at −80 °C. Patients with any obvious malignancies were excluded. Severity of AP was determined according to the revised Atlanta classification^1^. Serum samples were applied to a Bio-Plex Pro Human Chemokine Assay (BIO-RAD, Hercules, CA). The study was performed according to the Declaration of Helsinki, and was approved by the Institutional Review Board of Kyoto University Hospital. Written informed consent was obtained from all the participants before the entry to the study.

### CXCL16 measurement using a validation set of human sera

A validation set of human serum samples were obtained from patients with AP admitted to Saiseikai Nakatsu Hospital from 2016 to 2017, and from another set of healthy donors. Collection and storage of the sera were performed in the same manner as the screening set. The study for comprehensive analysis of serum cytokines/chemokines was also approved by the Institutional Review Board of Saiseikai Nakatsu Hospital and informed consent was obtained from all the participants. The study was performed according to the Declaration of Helsinki. Samples were applied to a human CXCL16 ELISA kit (R&D systems) and the levels of serum CXCL16 were measured according to the manufacturer’s instructions.

### Animals

*Cxcl16*^−/−^ mice backcrossed to C57BL/6 mice were maintained as described in our previous report^24^. C57BL/6 mice were purchased from Japan SLC (Shizuoka, Japan). All mice were fed standard laboratory chow and water *ad libitum*, and housed under specific pathogen-free conditions. All experiments were performed with mice at 8 weeks of age. All experiments were approved by the institutional animal ethics committee and performed according to the guidelines of the animal ethics committee of Kyoto University.

### Mouse pancreatitis model

Cerulein (Sigma-Aldrich, St. Louis, MO) was dissolved with phosphate-buffered saline (Wako, Osaka, Japan) and administered intraperitoneally at a dose of 100 µg/kg at 1-h intervals (total of 8 injections) for 1 or 2 consecutive days^28–30^. Five mice were sacrificed at the indicated time points. After the first cerulein injection, mice were maintained in a fasting state and water was provided *ad libitum*. To investigate the effect of Cxcl16 blockade on pancreatitis, mice were intraperitoneally injected with 450 μg of monoclonal anti-Cxcl16 Ab^24^ or control rat IgG (MP Biomedicals, Santa Ana, CA) dissolved in 200 μl of phosphate-buffered saline at 2 h after the 8th injection of cerulein on day 1 of the injections.

Serum amylase levels were determined by SPOTCHEM EZ (Arkray, Kyoto, Japan) according to the manufacturer’s instructions. For pathology scoring, pancreatic tissues were fixed in 4% formaldehyde and embedded in paraffin. Sections of 5-μm thickness were stained with hematoxylin and eosin (H&E), and five randomly selected high-power fields from each mouse were evaluated as previously described by Schmidt *et al*.^31^. For infiltrating cell counts, five randomly selected high-power fields of formaldehyde-fixed and paraffin-embedded sections from each mouse were evaluated using immunostaining of Gr1, F4/80, and CD3 for neutrophils, macrophages, and T cells, respectively.

### Quantitative RT-PCR

Total RNA was extracted from each mouse pancreas, mouse pancreatic acinar cells, or rat acinar cell line AR42J^32,33^, using RNeasy mini kit (Qiagen, Venlo, Netherlands). Single-strand cDNA was synthesized using a First Strand SYBR Green Master Mix (Roche Applied Science, Basel, Switzerland). Quantitative reverse transcription-PCR (qRT-PCR) was performed using FastStart SYBR Green Master (Roche Applied Science) and the LightCycler 480 system (Roche Applied Science). *Gapdh* was used as an internal control and the results were calculated by the comparative cycle threshold (Ct) method, with relative transcript levels determined by double-delta Ct. The primer sequences used for qRT-PCR are as follows:

mouse (m)*Gapdh*-forward, 5′-AGGTCGGTGTGAACGGATTTG-3′;

m*Gapdh*-reverse, 5′-TGTAGACCATGTAGTTGAGGTCA-3′;

m*Cxcl16*-forward, 5′-CGTTGTCCATTCTTTATCAGGTTCC-3′;

m*Cxcl16*-reverse, 5′-TTGCGCTCAAAGCAGTCCA-3′;

m*Tnf*α-forward, 5′-CAGGCGGTGCCTATGTCTC-3′;

m*Tnf*α-reverse, 5′-CGATCACCCCGAAGTTCAGTAG-3′;

m*Il6*-forward, 5′-CTGCAAGAGACTTCCATCCAGTT-3′;

m*Il6*-reverse, 5′-GAAGTAGGGAAGGCCGTGG-3′;

m*Cxcl2*-forward, 5′-AACATCCAGAGCTTGAGTGTGA-3′;

m*Cxcl2*-reverse, 5′-TTCAGGGTCAAGGCAAACTT-3′;

m*F4/80*-forward, 5′-CTGCACCTGTAAACGAGGCTT-3′;

m*F4/80*-reverse, 5′-GCAGACTGAGTTAGGACCACAA-3′;

m*Ccl9*-forward, 5′-CCCTCTCCTTCCTCATTCTTACA-3′;

m*Ccl9*-reverse, 5′-AGTCTTGAAAGCCCATGTGAAA-3′;

m*Vcam-1*-forward, 5′-TTGGGAGCCTCAACGGTACT-3′;

m*Vcam-1*-reverse, 5′-GCAATCGTTTTGTATTCAGGGGA-3′;

m*Ccl2*-forward, 5′-TTAAAAACCTGGATCGGAACCAA-3′;

m*Ccl2*-reverse, 5′-GCATTAGCTTCAGATTTACGGGT-3′;

rat (r)*Cxcl16*-forward, 5′-CGCTGTCAATTCTTTATCAGGTTCC-3′;

r*Cxcl16*-reverse, 5′-TTGTGCTCAAAGCAGTTCA-3′;

r*Ccl9*-forward, 5′-TGGGCCCACCAGGAGGATGAA-3′;

r*Ccl9*-reverse, 5′-TCTGTCGCATGTACGATCTGGGC-3′

### Immunohistochemistry

For immunostaining, the following antibodies were used as primary antibodies: rat monoclonal antibodies for Gr1 (eBioscience, San Diego, CA) and F4/80 (AbD Serotec, Raleigh, NC), and a rabbit monoclonal antibody for CD3 (Abcam plc, Cambridge, UK). Staining signals were detected using a Vectorstain Elite ABC Kit (Vector Laboratories, Burlingame, CA) and a peroxidase substrate kit, DAB (Vector Laboratories). For immunofluorescence staining, tissues were directly embedded in O.C.T. Compound (Sakura Finetek, Tokyo, Japan) and sectioned at a thickness of 6 μm. The sections were fixed with ice-cold methanol and immunostained using the following primary antibodies: goat polyclonal antibodies for Cxcl16 (R&D Systems, Minneapolis, MN) and Ccl9 (R&D Systems), a rabbit polyclonal antibody for amylase (Sigma-Aldrich). Antibodies for IgG labeled with Alexa Fluor 488 or Alexa Fluor 594 (Molecular Probes, Eugene, OR) were used as secondary antibodies. Nuclei were stained with Hoechst (Molecular Probes). Bright-field and fluorescence images were captured with an Olympus BX53-33FL microscope (Olympus, Tokyo, Japan). The captured images were analyzed with Adobe Photoshop software.

### Macrophage depletion

Macrophages were depleted by intraperitoneal injection of clodronate liposomes (100 mg/kg; Hygieia Bioscience, Osaka, Japan) at 24 h before the first cerulein injection. Macrophage depletion was confirmed by the decrease of F4/80 in immunostaining and mRNA expression in the spleen (data not shown).

### Cytokine/chemokine array in mice

Pancreatic lysates were prepared as described previously^16^. Briefly, pancreatic tissue was harvested and snap-frozen in liquid nitrogen. After thawing, the tissue was sonicated in lysis buffer (1% NP-40) supplemented with protease inhibitor cocktail (Sigma) and centrifuged at 12,000 g for 15 minutes at 4 °C, at which point the lysis supernatant was collected. Pancreatic lysates were applied to commercial membranes to which Mouse Cytokine Antibody Array 3 (RayBiotech, Norcross, GA) was bound for evaluation of cytokine and chemokine profiles. The relative expressions were measured using Image J software.

### ELISA

Levels of Ccl9 in the sera or the pancreatic lysates were measured using a mouse Ccl9 ELISA kit (R&D Systems), and levels of myeloperoxidase (MPO) in pancreatic lysates were determined using a mouse MPO ELISA kit (ThermoFisher Scientific, Hudson, NH), according to the manufacturer’s instructions.

### Cell culture and *in vitro* experiments

The procedures were carried out with sterile precautions, solutions, and apparatuses. AR42J cells^32,33^ were cultured in Dulbecco’s modified Eagle’s medium (DMEM)-low glucose (Sigma-Aldrich) complemented with 10% fetal bovine serum (FBS) and penicillin streptomycin antibiotics under 5% CO_2_. Cells were stimulated for 60 min with various concentrations of cerulein after 60-min preincubation with recombinant rat Cxcl16 (Sino Biological, Beijing, China), rat anti-Cxcl16 Ab^24^, or rat normal IgG (R&D Systems).

Freshly prepared pancreatic acinar cells were isolated from *WT* and *Cxcl16*^−/−^ mice as follows; pancreas was obtained from mice, dissected by small scissors, and then digested in 0.2 mg/ml collagenase-P (Roche) in a shaking water bath at 37 °C. Following two washes with cold DMEM supplemented with 10% FBS, digested pancreatic tissue was filtered through 100 μm nylon mesh (BD Falcon, Essex, UK). The cellular pellet was resuspended in DMEM supplemented with 10% FBS, 1000 U/ml penicillin G, 100 μg/ml streptomycin, and 0.1 mg/ml soybean trypsin inhibitor (Sigma), and stimulated for 60 min with 10^−7^ M cerulein. Samples that were not immediately processed were stored at −80 °C.

### Statistical analysis

Results were shown as mean ± SD. All statistical analyses were performed with JMP software version 11 (SAS, Cary, NC). Unpaired t test was used to evaluate the significance of the differences and a value of *p* < 0.05 was considered statistically significant, unless stated otherwise.

## Results

### CXCL16 is involved in the development of human severe acute pancreatitis

**Figure 1:**
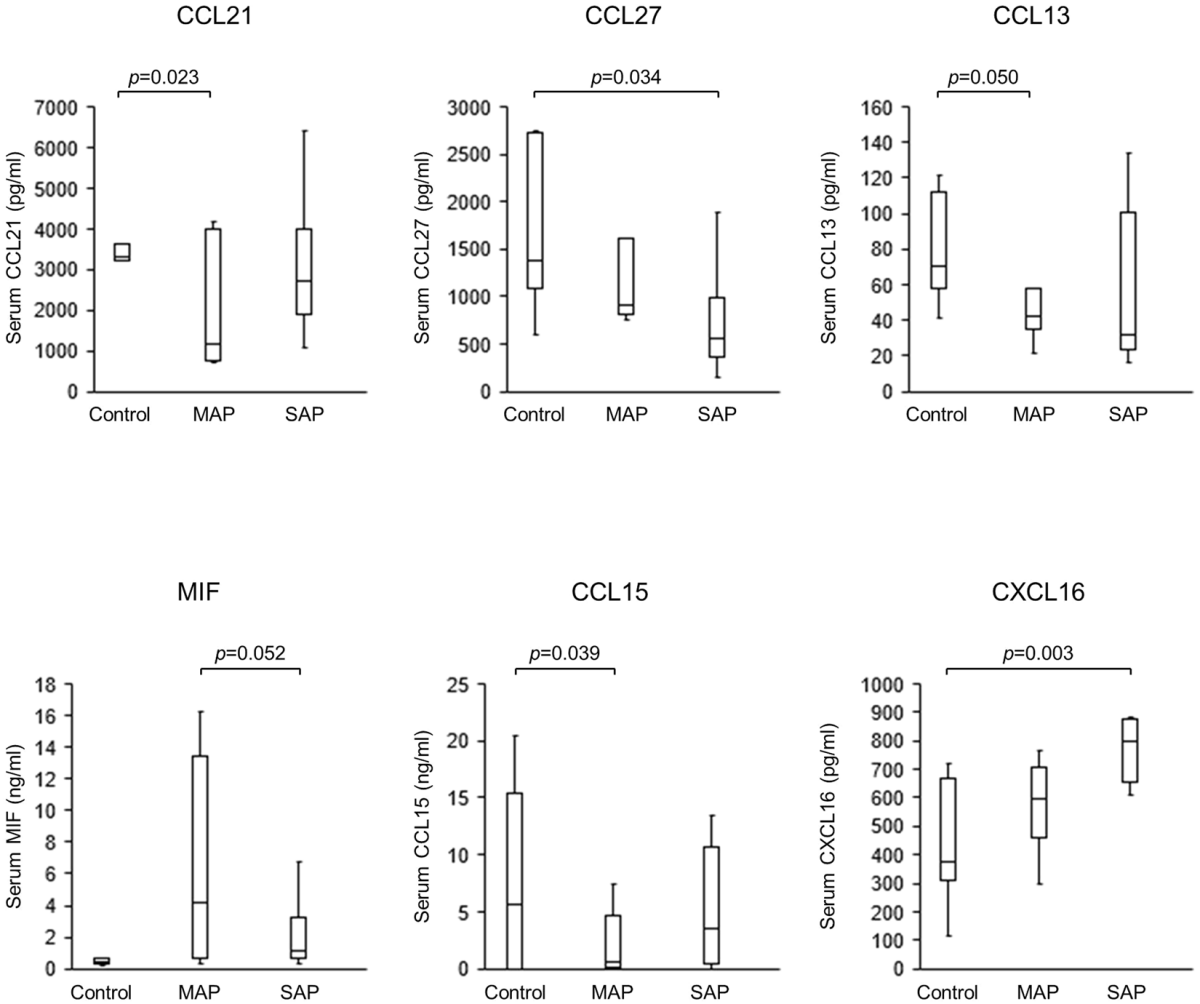
Serum cytokine/chemokine array in patients with AP. Serum levels of six chemokines, among 40 cytokines/chemokines investigated, were significantly altered in MAP and SAP patients. Serum levels of CCL21, CCL13, and CCL15 in MAP were significantly lower in patients than in controls. Serum levels of MIF were significantly lower in SAP patients than in MAP patients. Serum levels of CCL27 were significantly lower in SAP patients than in control patients. Serum levels of CXCL16 were significantly higher in SAP patients than in control patients. When Bonferroni method was adopted to correct multiple testing problem, only CXCL16 level was revealed to have a significant difference. MAP, mild acute pancreatitis; SAP, severe acute pancreatitis. Results were shown as mean ± SD.

We initially tried to identify cytokines and chemokines that play key roles in the development of SAP. To this end, we performed a cytokine/chemokine array by using serum samples obtained from patients with AP. We collected sera from patients with mild and severe AP according to the revised Atlanta classification^1^, and also from healthy controls. The patient characteristics are shown in Table 1. Serum lactate dehydrogenase values were significantly higher in SAP patients than in MAP patients or controls (*p* < 0.05), which may be due to greater tissue damage in SAP patients than in MAP patients. Six of eight SAP patients revealed impaired pancreatic vascular perfusion in contrast-enhanced computed tomography suggesting an involvement of pancreatic necrosis, but no patients received surgical operation or endoscopic/percutaneous drainage for infection. On the other hand, two of seven MAP patients exhibited only minimal perfusion abnormalities. Cytokine and chemokine array analysis using these AP patients revealed only 6 chemokines (CCL21, CCL27, CCL13, MIF, CCL15, CXCL16) of 40 cytokines/chemokines levels differed significantly among all comparisons between the three groups (MAP vs control, SAP vs control, and SAP vs MAP) (Fig. 1). Among these six candidate cytokines/chemokines, serum CXCL16 level increased in parallel to the severity of pancreatitis whereas such correlation between chemokine levels and the severity of pancreatitis was not seen in the other five chemokines. Thus, these data suggest that CXCL16 plays a role in the development of SAP.

**Table 1:** Clinical characteristics of patients with AP.

	Control n = 7	MAP n = 7	SAP n = 8
Age, year, mean (+/−SD)	62.6 (+/−13.7)	59.0 (+/−14.1)	59.0 (+/−19.9)
Sex, M/F	1/6	0/7	1/7
Serum amylase (IU/L)	109.2 (+/−40.8)	927.1 (+/−1129.6)	1079.1 (+/−995.54)^†^
Serum LDH (IU/L)	163.4 (+/−12.0)	200.1 (+/−52.7)	395.1 (+/−152.4)^†^*
Serum ALT (IU/L)	16.6 (+/−3.9)	25.7 (+/−16.7)	59.3 (+/−56.4)
Serum creatinine (mg/dL)	0.81 (+/−0.40)	0.56 (+/−0.19)	0.84 (+/−0.46)
PaO2 (mmHg)	—	81.1 (+/−6.7)	77.5 (+/−11.1)
Impairment of pancreatic perfusion in CECT	—	2/7	6/8
Severity mild/moderate/severe	—	7/0/0	0/6/2
Organ failure transient/persistent	—	0/0	2/2

^†^p < 0.05, compared with control; *p < 0.05, compared with mild pancreatitis;

MAP, mild acute pancreatitis; SAP, severe acute pancreatitis.

LDH, lactate dehydrogenase; ALT, alanine aminotransferase;

CECT, contrast-enhanced computed tomography.

Then we next aimed to validate the serum levels of CXCL16 in another set of samples. A total of 37 blood samples were obtained from 10 control people, 18 MAP patients, and 9 SAP patients. The patient characteristics are shown in Supplementary Table 1. Serum CXCL16 levels, determined by ELISA, were significantly higher in SAP patients than control or MAP patients (*p* < 0.01, Supplementary Fig. 1). Thus, CXCL16 elevation in the sera of patients with SAP was confirmed.

### Cxcl16 is involved in the development of necrotizing pancreatitis in mouse

**Figure 2:**
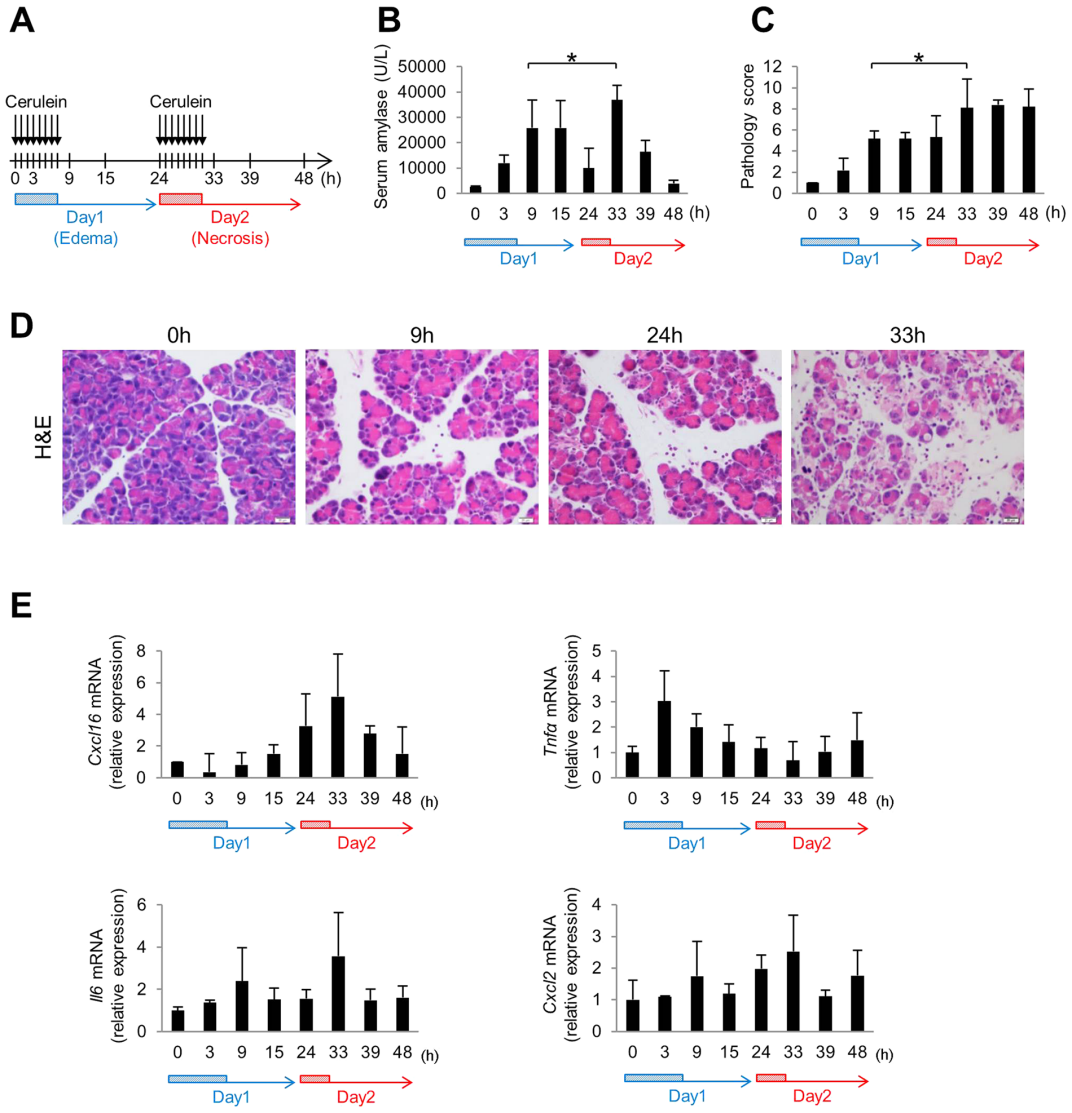
Cxcl16 expression in acute necrotizing pancreatitis. (**A**) Protocols of acute necrotizing pancreatitis by repeated injection of cerulein. Cerulein (100 ug/kg) was injected every 1 h 8 times on 2 consecutive days into C57BL/6 mice. Four to five mice were sacrificed at the indicated time points. (**B**) Serum amylase level and (**C**) pathology score of the respective time-points. (**D**) H&E sections in the pancreas of mice treated with acute necrotizing pancreatitis regimen at 0, 9, 24, and 33 h, respectively. The section at 33 h shows disrupted acinar architecture, vacuolization and necrosis of acinar cells, and inflammatory cell infiltration. (**E**) Pancreatic mRNA expressions of *Cxcl16*, *Tnfα*, *Il6*, and *Cxcl2* were determined by quantitative RT-PCR analysis. *p < 0.05 Results were shown as mean ± SD.

To investigate the causal relationship between the increase in serum CXCL16 levels and the progression of severe pancreatitis, we used a cerulein-induced mouse AP model. A series of eight hourly cerulein injections (Fig. 2A, edematous pancreatitis protocol) induced the mild edematous form of pancreatitis with increased serum amylase levels and pathologic changes but little acinar cell necrosis in 24 hours (Fig. 2B–D). Additionally, as described in previous reports^28–30^, two consecutive days of a series of eight cerulein injections (Fig. 2A, necrotizing pancreatitis protocol) induced a more severe necrotizing form of pancreatitis as judged by the serum amylase levels and the pathology scores (Fig. 2B–D). As shown in Fig. 2E, pancreatic expression of *Cxcl16* mRNA was markedly elevated in the late phase at 33 h on day 2 of this necrotizing pancreatitis model, but not early phase on day 1. Importantly, such an elevation of *Cxcl16* mRNA levels was parallel to serum levels of amylase and pathology scores on day 2. In contrast, mRNA expression of *Tnfα* was up-regulated at 3 h on day 1 in the early phase of AP and declined to the normal level on day 2. The mRNA expression of *Il6* or *Cxcl2*, a known neutrophil chemoattractant, showed bimodal peaks on day 1 and day 2 (Fig. 2E). Thus, CXCL16 was considered to be a candidate chemokine that determines the severity of pancreatitis since its expression was markedly up-regulated only in the late phase of acute pancreatitis with acinar cell necrosis.

These data suggest that progression of acinar cell necrosis in AP is associated with pancreatic expression of Cxcl16, a late phase chemokine.

### Cxcl16-deficient mice were resistant to the induction of acinar cell necrosis in AP

**Figure 3:**
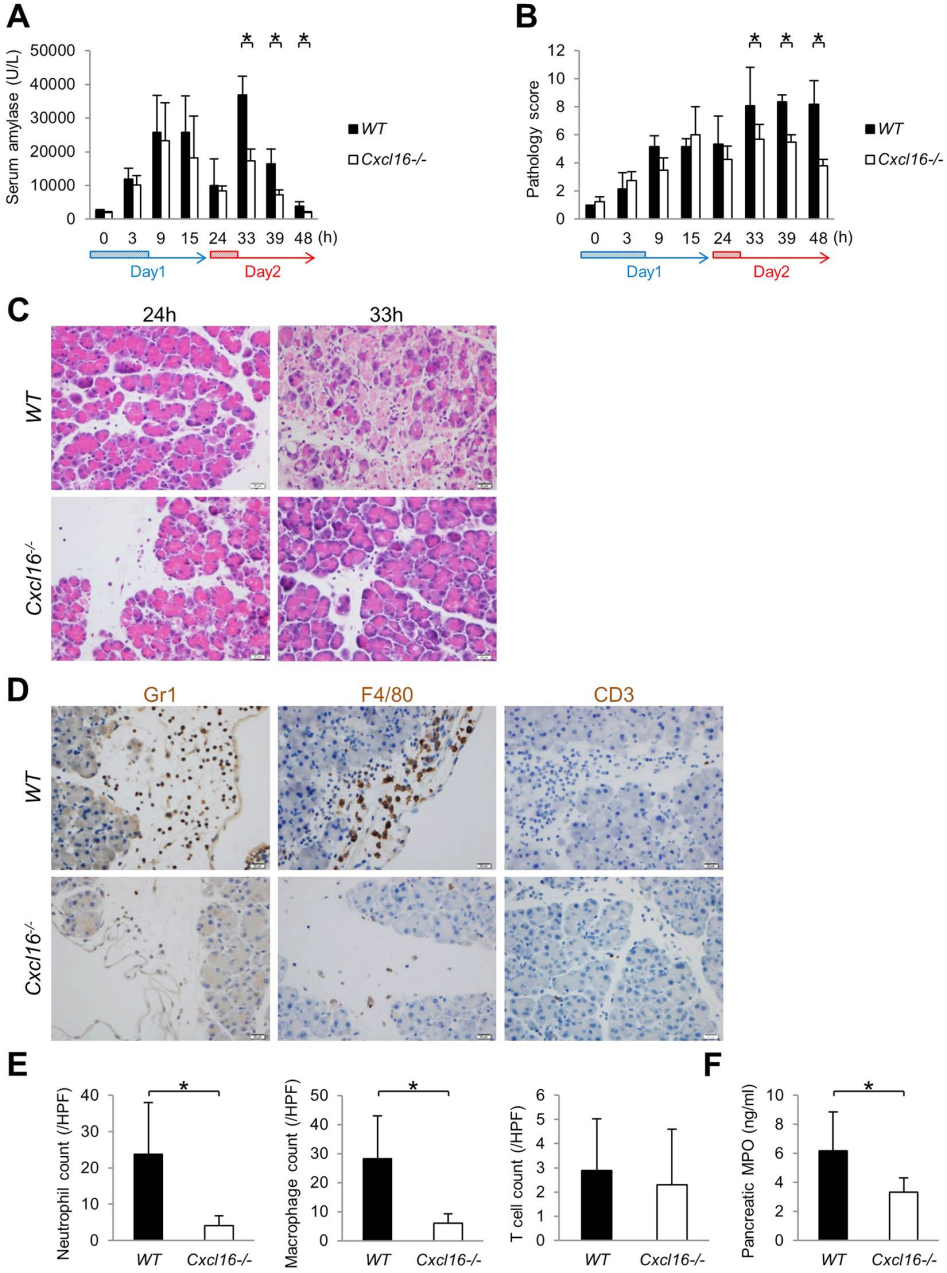
*Cxcl16*^−/−^ mice were resistant to the development of necrotizing pancreatitis. Cerulein (100 ug/kg) was injected into *Cxcl16*-intact (*WT*) and *Cxcl16*-deficient (*Cxcl16*^−/−^) mice (n = 5 in each group) every 1 h 8 times on 2 consecutive days as decribed in Fig. 2A. (**A**) Serum amylase levels and (**B**) pathology scores of *WT* and *Cxcl16*^−/−^ mice. (**C**) H&E sections from *WT* and *Cxcl16*^−/−^ mice at 24 h and 33 h. (**D**) Immunostained (Gr1, F4/80, and CD3) sections from *WT* and *Cxcl16*^−/−^ mice at 33 h. (**E**) Semiquantitative assessment of infiltartion of neutrophils, macrophages, and T cells. The number of neutrophils, macrophages, and T cells were counted in 5 high powered fields in each pancreas sections of *WT* and *Cxcl16*^−/−^ mice at 33 h. (**F**) MPO levels determined by ELISA in the pancreatic lysates of *WT* and *Cxcl16*^−/−^ mice at 33 h. *p < 0.05 Results were shown as mean ± SD.

To further investigate the role of Cxcl16 in the development of acinar cell necrosis in AP, we utilized *Cxcl16*-deficient (*Cxcl16*^−/−^) mice in our 2-day AP mouse model. We observed no significant differences in the serum amylase levels or pathology scores between *Cxcl16*-intact wild type (*WT*) and *Cxcl16*^−/−^ mice on day 1 in the development of edematous pancreatitis (Fig. 3A,B). In contrast, increased serum amylase levels and pathology scores observed in *WT* mice on day 2 were diminished in *Cxcl16*^−/−^ mice (Fig. 3A,B). Acinar cell necrosis, which developed in *WT* mice at 33 h on day 2, was not observed in *Cxcl16*^−/−^ mice while only edematous pancreatitis developed in *Cxcl16*^−/−^ mice (Fig. 3C). These data suggest that the progression of acinar cell necrosis requires intact Cxcl16-signaling pathways.

Since various kinds of immune cells such as neutrophils, macrophges, and T cells infiltrate into the inflamed pancreas^2–7^, we then turned our attention to the types of immune cells migrated into the pancreas in the presence or absence of Cxcl16. To this end, immuno-histochemical analysis was performed to visualize infiltration of Gr-1^+^ neutrophils, F4/80^+^ macrophages, and CD3^+^ T cells. As shown in Fig. 3D,E, pancreatic infiltration of Gr-1^+^ neutrophils and F4/80^+^ macrophages was markedly reduced in *Cxcl16*^−/−^ mice as compared with *WT* mice. In contrast, CD3^+^ T-cell infiltration level was comparable between the two groups (Fig. 3D,E). Consistent with a marked reduction of neutrophil infiltration in *Cxcl16*^−/−^ mice, pancreatic myeloperoxidase (MPO) was also significantly reduced in *Cxcl16*^−/−^ mice (Fig. 3F). Taken together, these studies employing *Cxcl16*-deficient mice strongly suggest that induction of Cxcl16 expression at a late phase of acute pancreatitis promotes the development of acinar cell necrosis through migration of myeloid cells into the inflamed pancreas.

### Cxcl16 is expressed in acinar cells in the development of necrosis in mice

**Figure 4:**
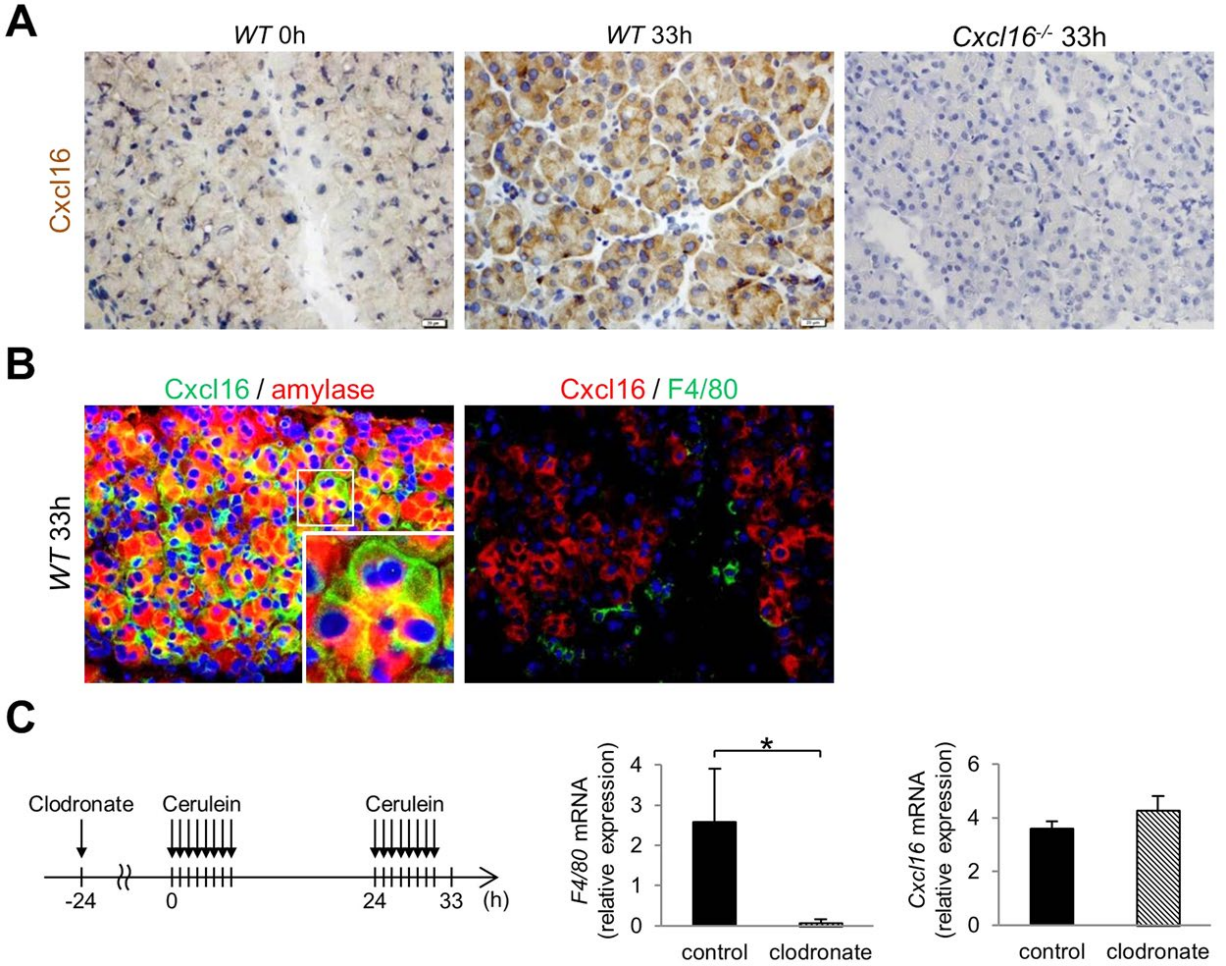
Acinar cell expression of Cxcl16. Cerulein (100 µg/kg) was injected into *WT* and *Cxcl16*^−/−^ mice every 1 h 8 times on 2 consecutive days as described in Fig. 2A. (**A**) Cxcl16 immunostaining of pancreatic frozen sections from *WT* mice (0 and 33 h) and *Cxcl16*^−/−^ mouse (33 h). (**B**) Dual immunofluorescence of Cxcl16 (Alexa Fluor 488) and amylase (Alexa Fluor 594), or Cxcl16 (Alexa Fluor 594) and F4/80 (Alexa Fluor 488). Most of Cxcl16-expressing on the cell surface was positive for cytoplasmic amylase expression. (**C**) Macrophage-depleted AP model (Left, protocol). Clodronate liposomes (100 mg/kg) were injected at 24 h before the first cerulein injection. *F4/80* and *Cxcl16* mRNA expression in the pancreas at 33 h, relative to those of control mice at 0 h, was evaluated at 33 h. *p < 0.05 Results were shown as mean ± SD.

Having obtained that Cxcl16 is a critical promoter for the development of acinar cell necrosis, we tried to identify the cellular source of Cxcl16. For this purpose, we performed immunohistochemical analysis in our 2-day AP mouse model. Strong Cxcl16 protein expression was induced in acinar cells of *Cxcl16*-intact *WT* mice at 33 h, whereas it was not detected at 0 h of *WT* or in *Cxcl16*^−/−^ mice (Fig. 4A). Double immunofluorescence staining also showed that the Cxcl16 expression was co-localized with amylase expression, but not with F4/80 expression, which suggests that Cxcl16 protein is expressed in amylase-expressing acinar cells, but not in F4/80-expressing macrophages (Fig. 4B). To further confirm the cellular source of Cxcl16 produced by acinar cells, depletion of macrophages by treatment with clodronate was performed. As shown in Fig. 4C, clodronate pre-treatment did not alter the expression level of pancreatic *Cxcl16* mRNA regardless of a marked reduction of pancreatic *F4/80* mRNA expression. These data suggest that acinar cells, but not myeloid cells, are the cellular source of Cxcl16.

### Decreased Ccl9 induction in acinar cells of *Cxcl16*^−/−^ mice

**Figure 5:**
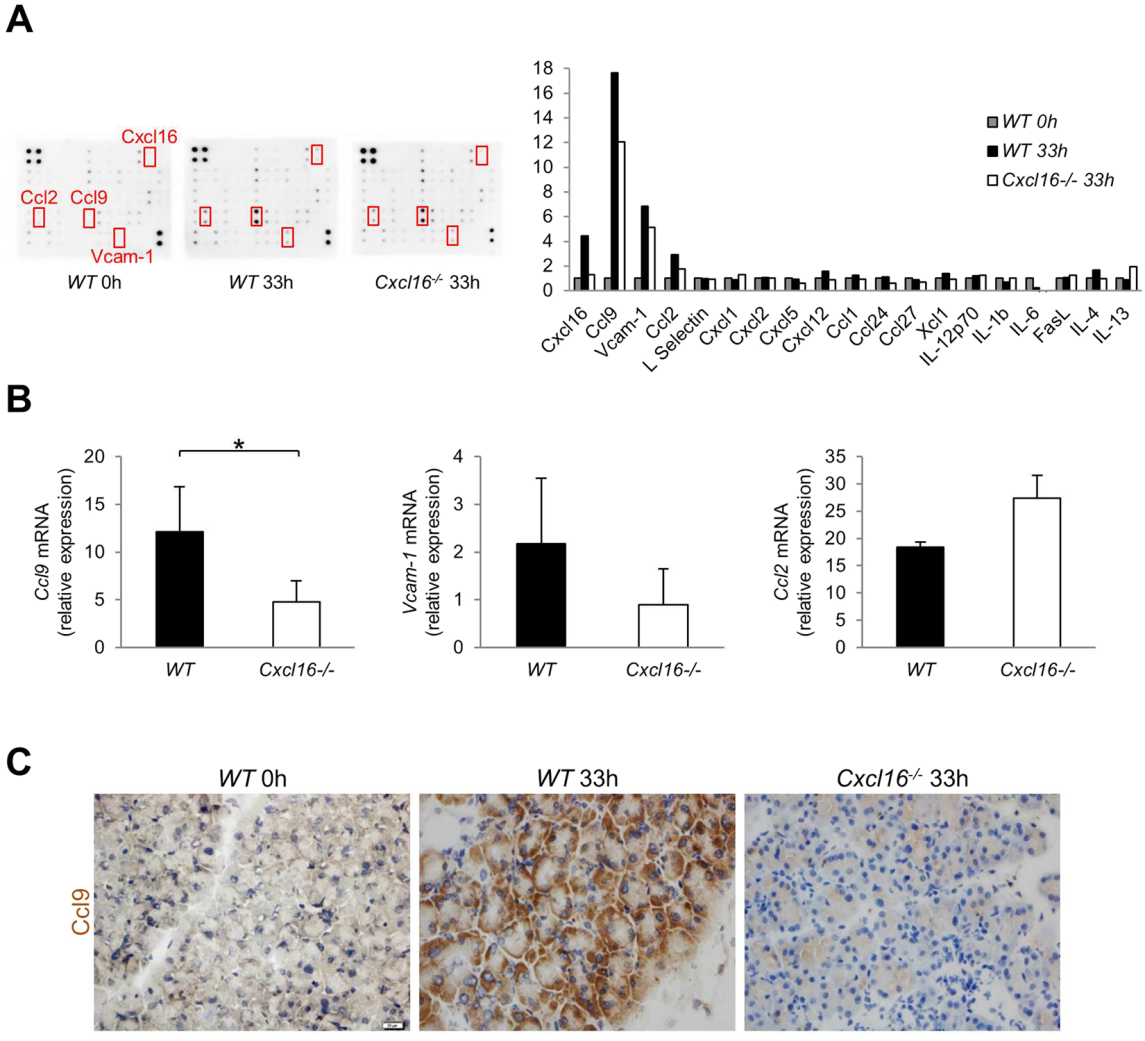
Induction of Ccl9 by Cxcl16 in necrotizing acute pancreatitis. (**A**) Cytokine/chemokine array using pancreatic lysates from C57BL/6 mice and *Cxcl16*^−/−^ mice treated with necrotizing pancreatitis regimen as described in Fig. 2A. The relative expression of cytokines and chemokines is shown together with representative images. The data presented were obtained from *WT* and *Cxcl16*^−/−^ mice at 33 h, and *WT* on 0 h. (**B**) *Ccl9*, *Vcam-1*, and *Ccl2* mRNA expression, relative to those of *WT* mice at 0 h, was assessed by qPCR in the pancreas of *WT* and *Cxcl16*^−/−^ mice at 33 h. (**C**) Ccl9 immunostaining of pancreatic frozen sections from *WT* mice at 0 h and 33 h, and *Cxcl16*^−/−^ mice at 33 h. *p < 0.05 Results were shown as mean ± SD.

Having confirmed that Cxcl16 plays a critical role in the development of acinar cell necrosis, we next tried to identify the down-stream effector molecule of Cxcl16. To this end, we performed a cytokine array using mouse pancreatic lysates from our 2-day AP mouse model at 0 h and 33 h. The array revealed a 4.5-fold induction of Cxcl16 protein at 33 h compared to that at 0 h (Fig. 5A). In addition, Ccl9, Vcam-1, and Ccl2 were also induced 17.6-, 6.8-, and 2.9-fold, respectively, at 33 h as compared to 0 h, while the levels of other cytokines were less than two-fold (Fig. 5A). The induction of all three soluble factors was reduced at 33 h in *Cxcl16*^−/−^ mouse as compared with *WT* mouse (Fig. 5A). Quantitative PCR analysis revealed that *Ccl9* mRNA expression was significantly lower in *Cxcl16*^−/−^ mice than in *WT* mice whereas expression of *Vcam-1* and *Ccl2* was comparable between these mice (Fig. 5B). Induction of Ccl9 expression was seen in acinar cells of *WT* mice at 33 h by immunohistochemical analysis. In contrast, such induction of Ccl9 expression was absent in *Cxcl16*^−/−^ mouse (Fig. 5C). Given the fact that Ccl9 is a potent neutrophil chemoattractant^34,35^, it is possible that Cxcl16 promotes the development of acinar cell necrosis through induction of Ccl9 and migration of neutrophils.

### Cxcl16 induces Ccl9 mRNA transcription in pancreatic acinar cells

**Figure 6:**
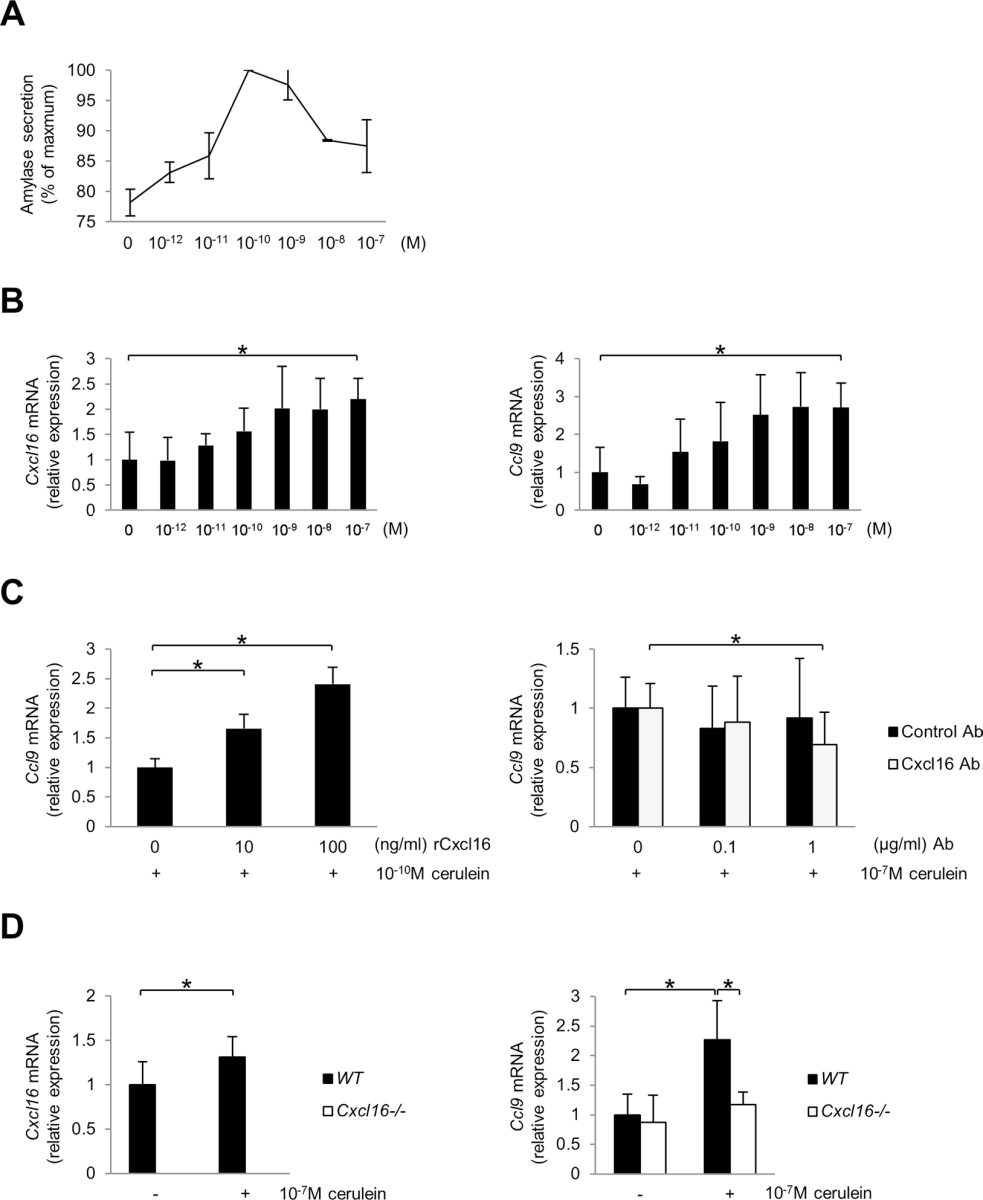
Expression of *Ccl9* by pancreatic acinar cells. (**A**) Amylase secretion by the rat acinar cell line AR42J upon stimulation with various concentrations of cerulein. (**B**) *Cxcl16* and *Ccl9* mRNA expression by AR42J upon stimulation with cerulein. (**C**) *Ccl9* mRNA expression of AR42J upon stimulation with cerulein (10^−10^ M) in combination with various concentrations of recombinant Cxcl16 protein (left). *Ccl9* mRNA expression of AR42J upon stimulation with cerulein (10^−7^ M) in the presence of neutralizing anti-Cxcl16 Ab or control Ab (right). (**D**) *Cxcl16* and *Ccl9* mRNA expression by pancreatic acinar cells isolated from *WT* and *Cxcl16*^−/−^ mice upon stimulation with cerulein (10^−7^ M). *p < 0.05 Results were shown as mean ± SD.

To investigate the direct association between Cxcl16 and Ccl9 in pancreatic acini, we applied a well-established *in vitro* cerulein hyperstimulation model using the AR42J pancreatic acinar cell line. As previously reported^36,37^, secretion of amylase by cerulein-stimulated acinar cells exhibited a bell-type dose response in which maximal secretion was seen at a physiologic dose (10^−10^ M) (Fig. 6A). Different from the case of amylase secretion, *Cxcl16* and *Ccl9* mRNA transcription were induced in a cerulein-dose dependent manner (Fig. 6B).

To clarify the relationship between *Cxcl16* and *Ccl9* mRNA induction by cerulein in AR42J cells, we next assessed the effect of recombinant Cxcl16 (rCxcl16) or neutralizing anti-Cxcl16 antibody treatment on *Ccl9* mRNA expression. Under a physiologic dose (10^−10^ M) of cerulein-stimulated condition, rCxcl16 treatment induced *Ccl9* mRNA transcription in AR42J cells in a dose-dependent manner (Fig. 6C left). In addition, the induction of *Ccl9* mRNA expression by a supraphysiologic dose (10^−7^ M) of cerulein was partially but significantly inhibited by anti-Cxcl16 Ab treatment (Fig. 6C right). These findings suggest that Cxcl16 promotes *Ccl9* mRNA expression in pancreatic acinar cells.

To further confirm the role of Cxcl16 in pancreatic acinar cells, we prepared freshly isolated acinar cells from *WT* and *Cxcl16*^−/−^ mice and stimulated with a high dose (10^−7^ M) of cerulein. As a result, cerulein treatment induced increase of *Cxcl16* and *Ccl9* mRNA expressions in *WT* acinar cells. In contrast, such an induction of *Ccl9* mRNA expression was not observed in *Cxcl16*^−/−^ acinar cells, confirming the role of Cxcl16 in Ccl9 expression in pancreatic acinar cells (Fig. 6D).

### Inhibition of Cxcl16 can prevent the development of necrotizing pancreatitis

**Figure 7:**
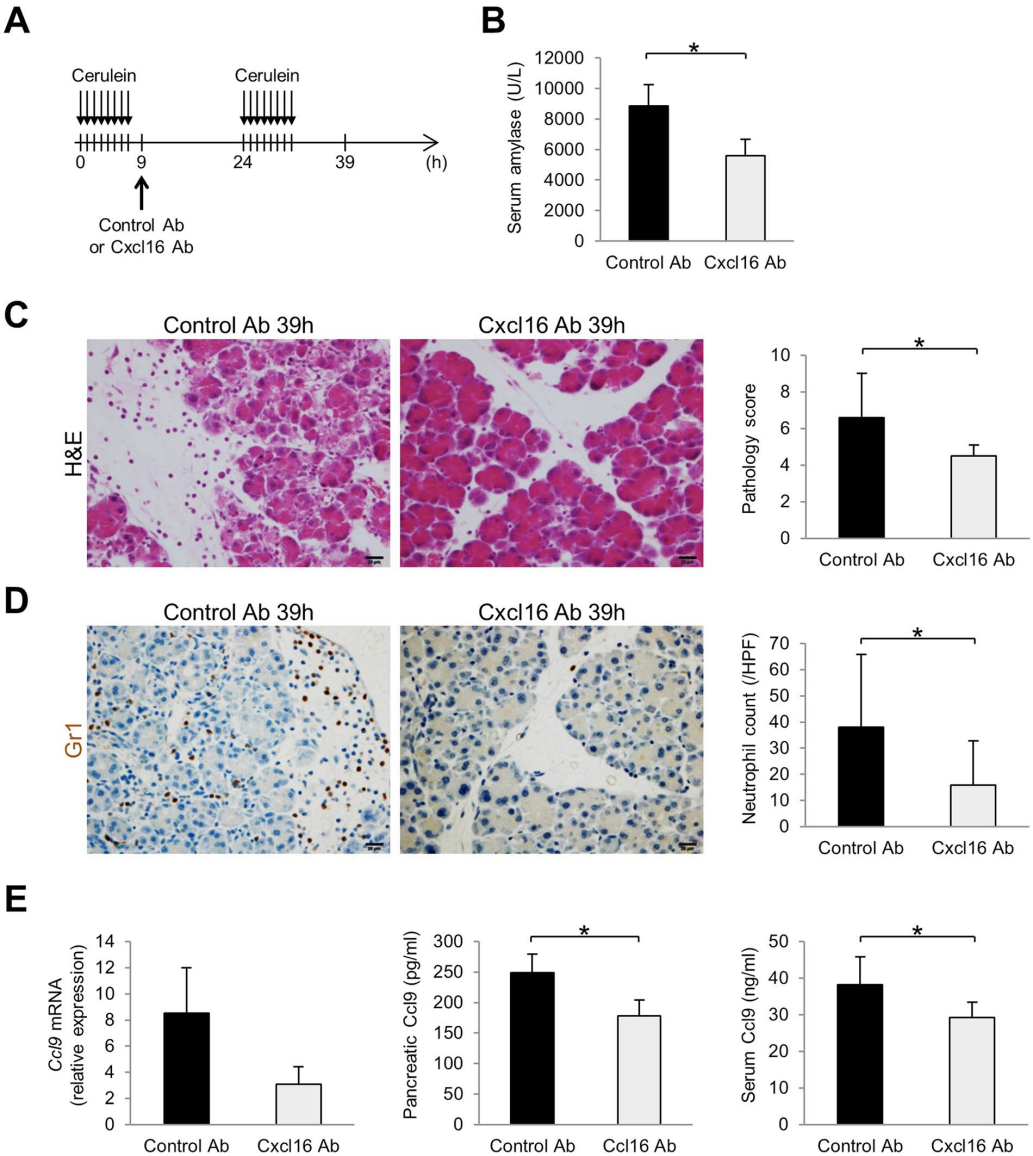
Therapeutic effects of neutralizing Cxcl16 Ab in the necrotizing pancreatitis model. (**A**) Injection protocol of anti-Cxcl16 Ab in necrotizing pancreatitis model. (**B**) Serum amylase level, (**C**) representative H&E sections, and pathology score of control Ab or anti-Cxcl16 Ab-treated mice at 39 h. (**D**) Gr1 immunostained sections and neutrophil counts of control Ab or anti-Cxcl16 Ab-treated mice at 39 h. (**E**) Pancreatic *Ccl9* mRNA, pancreatic Ccl9 protein, and serum Ccl9 protein levels in control Ab or anti-Cxcl16 Ab-treated mice at 39 h. n = 5 in each group. *p < 0.05 Results were shown as mean ± SD.

To test the therapeutic effect of Cxcl16 signaling blockade in AP, especially in the development of acinar cell necrosis, we administered anti-Cxcl16 Ab to our 2-day AP mouse model after the first series of 8 cerulein injections on day 1 (Fig. 7A). The serum amylase levels (Fig. 7B) and pathology scores (Fig. 7C) on the second day were significantly decreased in mice treated with anti-Cxcl16 Ab as compared with control mice. Histological examination revealed the development of necrotizing pancreatitis and edematous pancreatitis in mice treated with control Ab and anti-Cxcl16 Ab, respectively (Fig. 7C). Inhibition of development of acinar cell necrosis by anti-Cxcl16 Ab treatment was accompanied by a reduction in neutrophil infiltration (Fig. 7D), pancreatic *Ccl9* mRNA expression, pancreatic Ccl9 protein expression, and serum Ccl9 protein levels (Fig. 7E). Thus, these data suggest that Cxcl16 is a potential therapeutic target of necrotizing acute pancreatitis.

## Discussion

SAP has an extremely high mortality rate^1,2^. To improve the outcome of patients with the disease, in the present study, we focused on the mechanism underlying disease progression and severity. Based on an analysis of patient serum samples, mouse pancreatitis models, and *in vitro* experiments, we found that the chemokine CXCL16 is a key regulator in the development of acinar cell necrosis. Interestingly, Cxcl16 does not play a role as an acute phase pro-inflammatory mediator in the onset of AP, but rather this chemokine plays a critical role in the late phase of pancreatitis and accelerates inflammatory cascades through the induction of Ccl9 in mice. We also demonstrated that Cxcl16 could be a potential therapeutic target for acute pancreatitis.

To identify key molecules involved in the progression of acute pancreatitis in necrotizing forms, we first analyzed sera from AP patients using a cytokine/chemokine array. Since we tried to elucidate molecular mechanisms associated with the progression, but not the onset of the disease, we compared serum levels of cytokines and chemokines obtained from healthy controls and patients with mild or severe AP. Although the sample size is small, we successfully identified six candidate cytokines and chemokines that differed between groups. Notably, only serum CXCL16 levels had significant association with the severity of the pancreatitis and the relation was further confirmed in the validation set. Our results showed no evidence of infected necrosis, but are almost consistent with the previous report by Wittel *et al*. which revealed elevation of serum levels of CXCL16 in patients with SAP accompanied by infected pancreatic necrosis^18^. Thus, based on both our results and those of others, it seems likely that CXCL16 is highly associated with the development of necrotizing pancreatitis with or without infection.

To investigate the precise role of CXCL16 in the development of AP, we employed mouse AP progression model. As previously described^28–30^, two consecutive days of a series cerulein injections exacerbated AP on day 2 compared with day 1 based on the serum amylase levels and pathology scores. Our 2-day AP protocol successfully induced acinar cell necrosis on day 2, but did not increase serum LPS level (data not shown), which results were same as the previous report^30^. This mouse model is characterized by enhanced expression of acute phase cytokines such as Tnfα on day 1, recapitulating the cytokine expression pattern observed in AP patients^10,11^. Interestingly, *Cxcl16* mRNA expression was not increased on day 1, while it was markedly increased on day 2 in parallel with the increased serum amylase levels and pathology scores. Such expression pattern of Cxcl16 suggests that Cxcl16 is a molecular switch between edematous pancreatitis and necrotizing pancreatitis. This hypothesis was further supported by the results of our analysis of *Cxcl16*^−/−^ mice, showing that *Cxcl16*^−/−^ mice were resistant to the development of acinar cell necrosis on day 2 as compared with *Cxcl16*-intact mice, whereas the sensitivity to the induction of edematous pancreatitis on day 1 was comparable between both strains. Thus, we concluded that Cxcl16 is induced in the late phase of AP and contributes to the development of acinar cell necrosis.

Several reports show that macrophages and antigen presenting cells produce Cxcl16 in various pathologic conditions^21–23^. In the present study, however, immunohistochemistry clearly showed the induction of Cxcl16 expression in pancreatic acinar cells, but not in immune cells. Compatible to these results, depletion of macrophages did not alter the *Cxcl16* mRNA levels in pancreatic tissue, suggesting that pancreatic acinar cells are the cellular source of Cxcl16. Given the previous reports of Cxcl16 in epithelial cells including pancreatic cells^38^, it is reasonable that Cxcl16 in pancreatic acinar cells plays a role in the progression of AP as is the case of other chemokines^13–16,39^. For example, CCL2 produced by acinar cells leads to the development of pancreatitis via migration of CCR2-expressing myeloid cells into the pancreas^14–16^. In our study, migration of neutrophils and macrophages was markedly decreased in *Cxcl16*^−/−^ mice as compared with *Cxcl16*-intact mice. Therefore, Cxcl16 expression, which is induced at a late phase of acute pancreatitis on day 2, leads to the development of acinar cell necrosis through recruitment of myeloid cells into the pancreas.

As for the molecular mechanisms by which acinar cells–derived Cxcl16 mediate pancreatic infiltration of myeloid cells, we have clarified a role played by Ccl9. The mRNA expression of *Ccl9*, a neutrophil chemoattractant^34,35^, was significantly lower in the pancreas of *Cxcl16*^−/−^ mice as compared with *Cxcl16*-intact mice. Ccl9 was also expressed in pancreatic acinar cells of mice treated with our 2 consecutive day cerulein protocol. In addition, pancreatic acinar cells produce a large amount of Ccl9 in response to simultaneous stimulation with cerulein and Cxcl16. Based on these results, we assume that Cxcl16 induces Ccl9 in pancreatic acinar cells in an autocrine manner, and that this Cxcl16/Ccl9 cascade plays a critical role in the development of acinar cell necrosis, through the recruitment of neutrophils. Neutrophil-derived MPO, elastase, reactive oxygen species, and neutrophil extracellular traps are reported to be involved in the progression of acinar cell necrosis in AP^4–6,12,40^. The observation of a lower neutrophil count and lower pancreatic MPO in *Cxcl16*^−/−^ mice is consistent with this idea. Such intra-acinar autocrine signaling through Cxcl16 induced by inflammatory stimuli was also reported by Chalabi-Dchar *et al*.^38^. In addition, some recent reports show that CXCL16 itself could be a chemoattractant for myeloid cells^41,42^.

Finally, we evaluated the potential of Cxcl16 as a therapeutic target of AP. As emphasized above, Cxcl16 is not an acute-phase chemokine involved in the onset of AP, but rather a key promoter in the progression of SAP in the late-phase. To address this unique characteristic of Cxcl16, a neutralizing Ab against Cxcl16 was administered after the induction of AP on day 1 to prevent aggravation of the disease on day 2 in our experiments. Importantly, progression of AP with the development of acinar cell necrosis was significantly ameliorated by this treatment as judged by both the serum amylase levels and pathology scores, which effects were accompanied by a reduction in serum Ccl9 levels, pancreatic Ccl9 expression, and the degree of neutrophil infiltration. Given the fact that patients with AP are generally hospitalized after the onset of the disease, it is reasonable that a late-phase exacerbating factor such as Cxcl16 in this study rather than acute phase cytokines/chemokines could be a therapeutic target to prevent the progression of the disease after hospitalization.

In conclusion, chemokine CXCL16 is involved in the development of acinar cell necrosis. Our *in vivo/in vitro* data provide evidence that Cxcl16 is induced in pancreatic acinar cells and contributes to the development of acinar cell necrosis by activating Ccl9 and subsequent neutrophil recruitment. This late-phase exacerbating factor of AP could be a therapeutic target to prevent the progression of the disease.

## Electronic supplementary material

Supplementary Information
