# Supplementary material for: Chemokine CXCL16 mediates acinar cell necrosis in cerulein induced acute pancreatitis in mice
Source: Sci Rep. 2018 Jun 11;8:8829. doi: 10.1038/s41598-018-27200-y (PMC5995899; doi:10.1038/s41598-018-27200-y)
Supplement: Supplementary file 1 — Supplementary Information [file 41598_2018_27200_MOESM1_ESM.pdf]

Supplementary information

Chemokine CXCL16 mediates acinar cell necrosis in cerulein induced acute pancreatitis in mice

Yojiro Sakuma,[1] Yuzo Kodama,[1] Takaaki Eguchi,[2] Norimitsu Uza,[1] Yoshihisa Tsuji,[1, 3] Masahiro Shiokawa,[1] Takahisa Maruno,[1] Katsutoshi Kuriyama,[1] Yoshihiro Nishikawa,[1] Yuki Yamauchi,[1] Motoyuki Tsuda,[1] Tatsuki Ueda,[1] Tomoaki Matsumori,[1] Toshihiro Morita,[1] Teruko Tomono,[1] Nobuyuki Kakiuchi,[1] Atsushi Mima,[1] Yuko Sogabe,[1] Saiko Marui,[1] Takeshi Kuwada,[1] Akihiko Okada,[2] Tomohiro Watanabe,[1, 4] Hiroshi Nakase,[1, 5] Tsutomu Chiba,[1, 6] Hiroshi Seno[1]

[1]Department of Gastroenterology and Hepatology, Graduate School of Medicine, Kyoto University, Kyoto, Japan

[2]Department of Gastroenterology and Hepatology, Saiseikai Nakatsu Hospital, Osaka, Japan

[3]Department of Clinical Education, Shiga University of Medical Science, Otsu, Japan

[4]Department of Gastroenterology and Hepatology, Kindai University Faculty of Medicine, Osaka-Sayama, Japan

[5]Department of Gastroenterology and Hepatology, Sapporo Medical University School of Medicine, Sapporo, Japan

[6]Kansai Electric Power Hospital, Osaka, Japan

Supplementary Table 1. Clinical characteristics of patients in the validation analysis.

	Control<br>n=10	MAP<br>n=18	SAP<br>n=9
Age, year, mean (+/-SD)	67.6 (+/-16.6)	69.2 (+/-18.7)	69.7 (+/-18.1)
Sex, M/F	5/5	12/6	4/5
Serum amylase (IU/L)	65.7<br>(+/-25.3)	877.3<br>(+/-891.1)	1824.6<br>(+/-2262.4)†
Serum LDH (IU/L)	199.7<br>(+/-50.5)	283.9<br>(+/-179.4)	358.1<br>(+/-220.8)
Serum ALT (IU/L)	20.3 (+/-12.9)	135.3 (+/-151.3)	88.9 (+/-108.1)
Serum creatinine (mg/dL)	0.78 (+/-0.21)	0.72 (+/-0.25)	0.86 (+/-0.42)
PaO2 (mmHg)	-	87.7 (+/-12.2)	73.4 (+/-16.8)*
Impairment of pancreatic perfusion in CECT	-	0/18	2/9
Severity mild/moderate/severe	-	18/0/0	0/4/5
Organ failure transient/persistent	-	0/0	0/5

† $p<0.05$, compared with control; * $p<0.05$, compared with mild pancreatitis;

MAP, mild acute pancreatitis; SAP, severe acute pancreatitis;

LDH, lactate dehydrogenase; ALT, alanine aminotransferase;

CECT, contrast-enhanced computed tomography

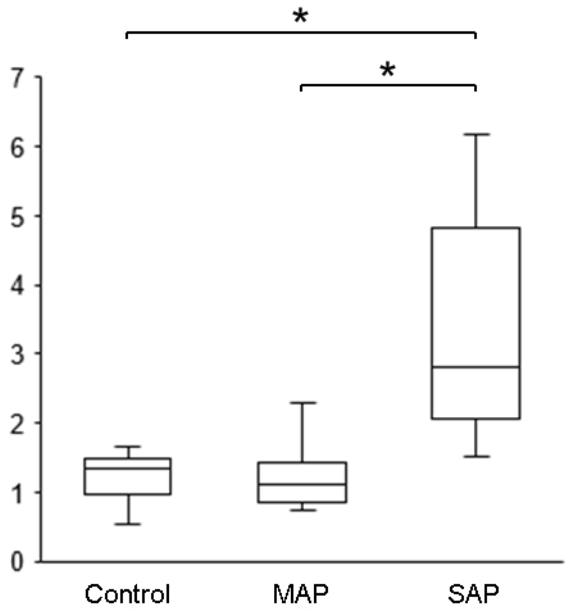

Supplementary Figure 1. Serum CXCL16 levels in the validation set of AP patients.

Serum levels of CXCL16 were measured by ELISA, using blood samples from 10 healthy donors, 18 MAP patients, and 9 SAP patients. Serum level of CXCL16 was significantly higher in SAP patients than MAP patients or controls. ** p<0.01
